# Elevated IL-6 plasma levels are associated with GAD antibodies-associated autoimmune epilepsy

**DOI:** 10.3389/fncel.2023.1129907

**Published:** 2023-03-21

**Authors:** Pabitra Basnyat, Maria Peltola, Jani Raitanen, Suvi Liimatainen, Sirpa Rainesalo, Marko Pesu, Jukka Peltola

**Affiliations:** ^1^Department of Neurology, Faculty of Medicine and Health Technology, Tampere University, Tampere, Finland; ^2^Department of Neurology, Tampere University Hospital, Tampere, Finland; ^3^Department of Psychiatry, Tampere University Hospital, Tampere, Finland; ^4^Faculty of Social Sciences (Health Sciences), Tampere University, Tampere, Finland; ^5^UKK Institute for Health Promotion Research, Tampere, Finland; ^6^Administration Centre, Tampere University Hospital, Tampere, Finland; ^7^Division of Acute Treatment, Emergency Department, Intensive Care and Anesthesia, Tampere University Hospital, Tampere, Finland; ^8^Laboratory of Immunoregulation, Faculty of Medicine and Health Technology, Tampere University, Tampere, Finland; ^9^Fimlab Laboratories, Tampere, Finland; ^10^Gilead Sciences, Vantaa, Finland

**Keywords:** epilepsy, autoimmunity, cytokines, GAD antibodies, IL-6, IL-10

## Abstract

**Background:**

Antibodies against glutamic acid decarboxylase (GADA) are present in multiple neurological manifestations, such as stiff-person syndrome, cerebellar ataxia, limbic encephalitis, and epilepsy. Increasing data support the clinical significance of GADA as an autoimmune etiology of epilepsy, however, there is not yet definitive evidence to confirm the pathogenic link between GADA and epilepsy.

**Objective:**

Interleukin-6 (IL-6), a pro-convulsive and neurotoxic cytokine, and interleukin-10 (IL-10), an anti-inflammatory and neuroprotective cytokine, are crucial inflammatory mediators in the brain. Increased production of IL-6 and its association with epileptic disease profiles are well established, suggesting the presence of chronic systemic inflammation in epilepsy. Therefore, in this study, we investigated the association of plasma cytokine concentrations of IL-6 and IL-10 and their ratio with GADA in patients with drug-resistant epilepsy.

**Methods:**

Interleukin-6 and IL-10 concentrations were measured by ELISA in plasma, and the IL-6/IL-10 ratio was calculated in a cross-sectional cohort of 247 patients with epilepsy who had their GADA titers measured previously for their clinical significance in epilepsy. Based on GADA titers, patients were grouped as GADA negative (*n* = 238), GADA low positive (antibody titers < 1,000 RU/mL, *n* = 5), and GADA high positive (antibody titers ≥ 1,000 RU/mL, *n* = 4).

**Results:**

Median IL-6 concentrations were significantly higher in patients with high GADA positivity [2.86 pg/mL, interquartile range (IQR) = 1.90–5.34 pg/mL] than in GADA-negative patients [1.18 pg/mL, interquartile range (IQR) = 0.54–2.32 pg/mL; *p* = 0.039]. Similarly, IL-10 concentrations were also higher in GADA high-positive patients [1.45 pg/mL, interquartile range (IQR) = 0.53–14.32 pg/mL] than in GADA-negative patients [0.50 pg/mL, interquartile range (IQR) = 0.24–1.00 pg/mL], however, the difference was not statistically significant (*p* = 0.110). Neither IL-6 nor IL-10 concentrations were different between GADA-negative and GADA low-positive patients (*p* > 0.05) or between GADA low-positive or GADA high-positive patients (*p* > 0.05). The IL-6/IL-10 ratio was also similar among all the study groups.

**Conclusion:**

Increased circulatory concentrations of IL-6 are associated with high GADA titers in patients with epilepsy. These data provide additional pathophysiological significance of IL-6 and help to further describe the immune mechanisms involved in the pathogenesis of GADA-associated autoimmune epilepsy.

## 1. Introduction

Autoantibodies against glutamic acid decarboxylase (GADA) are present in 80% of patients with autoimmune type 1 diabetes mellitus (T1DM) and are used as diagnostic markers for T1DM (Sigurdsson and Baekkeskov, 1990; Solimena and De Camilli, 1991). These antibodies are directed against secretory vesicles in pancreatic islet cells, resulting in damage to insulin-producing pancreatic cells, and an important mechanism for the development of T1DM. In addition to T1DM, GADA has been detected in patients with multiple neurological manifestations, including stiff-person syndrome (SPS), cerebellar ataxia, limbic encephalitis, and epilepsy (Munoz-Lopetegi et al., 2020). In the brain, GADA is directed against glutamic acid decarboxylase, the enzyme that is important for the formation of the principal inhibitory neurotransmitter gamma-aminobutyric acid (GABA) and therefore impairs GABA synthesis. There are two different isoforms of GAD that are generated from two different genes, GAD65 and GAD67.

Increasing evidence supports the clinical importance of GADA and other autoantibodies in epilepsy, providing an immunologic basis for the autoimmune etiology of epilepsy (Peltola et al., 2000; Ranua et al., 2004; McKnight et al., 2005; Liimatainen et al., 2010). A high prevalence of GADA is often associated with temporal lobe epilepsy (TLE); therefore, GADA is considered one of the immunological markers of drug-resistant TLE (Falip et al., 2012). Previously, we detected high titers of GADA in epilepsy patients, and almost 90% of them were patients with TLE (Liimatainen et al., 2010). Moreover, we have shown that early detection of GAD65 antibodies facilitates the effectiveness of immunotherapy in patients with drug-resistant epilepsy (DRE) (Mäkelä et al., 2018).

Cytokines and autoantibodies have a critical role in the pathogenesis of several autoimmune diseases (Moudgil and Choubey, 2011). In drug-resistant focal epilepsy, especially in TLE, the association of proinflammatory cytokines, particularly interleukin-6 (IL-6), with epileptic disease profiles has been well established (Liimatainen et al., 2013). IL-6 is a pro-convulsive and neurotoxic cytokine that stimulates the production of most acute phase proteins. A variety of immune cells produce IL-6, but the most important ones are macrophages and monocytes at sites of inflammation. In our previous studies, we detected chronically increased serum concentrations of IL-6 in TLE patients compared with those in healthy controls (Liimatainen et al., 2009); these patients also exhibited a postictal increase in plasma IL-6 (Alapirtti et al., 2009). Furthermore, we also observed increased plasma IL-6 in TLE patients compared to extratemporal lobe epilepsy (XLE) patients, suggesting that the epilepsy type is important for determining seizure-induced IL-6 production (Alapirtti et al., 2018), and recently, we observed that IL-6 plasma levels are modified by hippocampal sclerosis (HS) and its lateralization in drug-resistant temporal lobe epilepsy (accepted). Interleukin-10 (IL-10), an anti-inflammatory and neuroprotective cytokine, has been associated with the immunopathology of several diseases, but its role in epilepsy is less studied (Basnyat et al., 2020). In the brain, it inhibits the production of proinflammatory cytokines by microglia, promotes the production of transforming growth factor-β by astrocytes, promotes neuronal cell survival and regulates adult neurogenesis (Lobo-Silva et al., 2016).

The purpose of this study was to analyses the IL-6 and IL-10 concentrations, including the IL6/IL10 ratio, with regard to GADA titers in the same patient cohort that was previously evaluated for the clinical significance of GAD antibodies in epilepsy (Liimatainen et al., 2010).

## 2. Materials and methods

### 2.1. Patients

This cross-sectional study comprises 253 consecutive adult patients with drug-resistant epilepsy treated at the Outpatient Clinic of Neurology of Tampere University Hospital. The Ethics Committee approved the study protocol of Tampere University Hospital, and all the patients provided written informed consent according to the Declaration of Helsinki. Epilepsy was classified according to the International League Against Epilepsy (ILAE) guidelines (Scheffer et al., 2017). Epilepsy types were categorized into TLE, frontal lobe, parietal lobe, occipital lobe, multifocal, and unknown focal epilepsies, and idiopathic generalized epilepsy (IGE). Patients with TLE were further assigned to either the TLE with HS group or the TLE without HS group, depending on the presence of HS. DRE was defined as persistent seizures after administering two different anti-seizure medications (ASMs) with adequate dosing (Kwan et al., 2010). Patients with DRE in our study population were evaluated for the possibility of epilepsy surgery. Patients with dementia, moderate or severe intellectual disability or malignant brain tumors were excluded from the study. Data regarding concomitant autoimmune diseases were collected. Due to limited access and practical difficulties in obtaining a meaningful healthy control group, the study lacks cytokines measurement in the healthy population. Therefore, we were unable to compare the cytokines levels between epilepsy patients and healthy control group.

### 2.2. Detection of GADA

Glutamic acid decarboxylase in sera was analyzed by radioimmunoassay (RIA) as previously described (Savola et al., 1998) in the Scientific Laboratory, Hospital for Children and Adolescents, University of Helsinki, Helsinki, Finland. When the cut-off limit for high GADA titers was defined based on patients with positive GADA levels, two distinct patient groups were identified: the first with high titers (≥1,000 RU/ml) and the second with low titers (<1,000 RU/ml) without associated autoimmune diseases. The cut-off limit for GADA positivity was 5.36 RU/ml as described previously (Liimatainen et al., 2010). The positive findings were validated by immunohistochemistry and immunoblotting of recombinant human GAD65. A detailed explanation related to the calculation of the cut-off limit for GADA positivity was provided previously (Liimatainen et al., 2010).

### 2.3. IL-6 and IL-10 measurements

Blood samples were collected during scheduled outpatient visits. Blood was collected in a vacutainer EDTA vacuum tube and centrifuged at 3,000 rpm for 10 min, and the separated plasma samples were frozen and stored at −70°C until use. Plasma IL-6 and IL-10 concentrations were measured using commercially available enzyme-linked immunosorbent assay (ELISA) kits according to the manufacturer’s protocol (Pelikine^®^ Compact, Sanquin, Amsterdam, Netherlands).

### 2.4. Statistical analysis

Clinical characteristics of the patients were presented as the means and standard deviations (SD) or frequencies and percentages. Continuous variable data were analyzed using the non-parametric Kruskal-Wallis H test or Mann–Whitney U test. When examining an association between groups and categorized variables, Pearson’s chi-squared test, if assumptions were valid, or Fisher’s exact test was used. Spearman’s correlation coefficient was used to analyse the correlation between the cytokines and their ratio with the frequency of seizures last month before the lab. The distribution of IL-6 levels, IL-10 levels, and IL-6/IL-10 ratio was visualized using scattergrams. All statistical analyses were performed using Stata statistical software version 17.0 (StataCorp, College Station, TX, USA). The *p*-values were considered significant at *p* ≤ 0.05. Figures were prepared using GraphPad Prism 5.02 software (GraphPad Software Inc., La Jolla, CA, USA).

## 3. Results

### 3.1. Patients

A total of 253 epilepsy patients (135 females and 118 males) with a median age of 39.0 years (range: 16–76) were enrolled in this study. The clinical characteristics were described in more detail in our previous publications (Liimatainen et al., 2010; Basnyat et al., 2020). Six patients had missing IL-6 and IL-10 measurement, therefore only 247 patients who had both GADA and cytokine measurements were included in the analyses. Groups based on GADA titers included a first GADA-negative or normal group, a second GADA low-positive group (antibody titers < 1,000 RU/mL, *n* = 5), and a third GADA high-positive group (antibody titers ≥ 1,000 RU/mL, *n* = 4, Table 2). The number of autoimmune diseases in the GADA high-positive group was higher than that in the GADA-negative group (57.1% vs. 7.6%, *p* = 0.030). The classification of patients based on GADA levels and their clinical characteristics is presented in Table 1. A summary of previous GADA findings updated with current cytokine findings are presented in Table 2.

**TABLE 1:** Background characteristics, i.e., mean and standard deviation for age, age at diagnosis, and duration of epilepsy or frequency and percentage for other variables.

	GADA-negative	GADA low-positive	GADA high-positive	*P*-value
N*	238	8	7	
Female	125 (52.5)	5 (62.5)	5 (71.4)	0.61^1^
Age, years	38.5 (14.3)	42.8 (15.9)	49.6 (16.9)	0.16^2^
Age at onset, years	16.4 (13.1)	23.4 (14.7)	17.3 (11.0)	0.20^2^
Type of epilepsy				0.15^1^
TLE	123 (51.7)	2 (25.0)	6 (85.7)	
Other focal	83 (34.9)	5 (62.5)	0	
IGE	32 (13.4)	1 (12.5)	1 (14.3)	
Etiology				0.89^1^
Structural	136 (57.1)	5 (62.5)	3 (42.9)	
Unknown	72 (30.3)	2 (25.0)	3 (42.9)	
Genetic	30 (12.6)	1 (12.5)	1 (14.3)	
Duration of epilepsy, years	22.2 (14.6)	19.5 (18.0)	32.1 (26.6)	0.60^2^
At least one seizure 1 year before lab	194 (81.9)	7 (87.5)	6 (85.7)	1.00^1^
Autoimmune diseases	18 (7.6)	0	4 (57.1)	**0.002** ^1^
Surgery				0.77^1^
No surgery	199 (83.6)	8 (100.0)	6 (85.7)	
Epilepsy surgery	18 (7.6)	0	1 (14.3)	
Other lesional surgery	21 (8.8)	0	0	
MRI is abnormal	121 (55.0)	5 (62.5)	3 (42.9)	0.78^1^

^1^ Fisher’s exact test; ^2^ Kruskal–Wallis H test.

IGE, idiopathic generalized epilepsy; TLE, temporal lobe epilepsy; *Six patients had missing cytokine measurement, therefore they are not included in the table. Bold values represent significant *P*-values.

**TABLE 2:** Clinical characteristics and median cytokine levels of GADA positive patients.

Patient number	Sex	Age	Epilepsy type	Etiology of epilepsy	Autoimmune diseases	GADA S-RIA (RU/ml)	IS of GADA	Other autoantibodies	IL-6 (pg/mL)	IL-10 (pg/mL)	IL-6/IL-10 ratio
1	F	32	TLE	Unknown	Thyroiditis, psoriasis	38,138	No	TPO, TYGL, IgM GM1, LANG	1.66	1.15	1.44
2	M	71	TLE	HS	No	8,534	Not done	TPO, IgG GM1, LANG	2.63	0.33	7.85
3	F	31	TLE	Unknown	CD, IgA deficiency, juvRA	4,569	Yes	ANA, IgG GM1	6.10	18.51	0.33
4	F	45	TLE	Immune	T1D, demyelinating disease	1,680	Yes	TPO, β2GP1, IgM GM1, IgG GM1	3.09	1.76	1.76
5	F	58	TLE	HS	No	800	No	AGPC, IgM GM1	1.69	1.97	0.86
6	F	60	FLE	Trauma	No	14.1	No	No antibodies	0.32	0.21	1.54
7	F	40	TLE	HS	No	10.9	No	No antibodies	0.27	0.53	0.52
8	M	37	FLE	Trauma	No	6.4	No	No antibodies	0.46	0.33	1.39
9	M	48	FLE	Unknown	No	6.3	No	IgG GM1	1.76	0.00	0.00

F, female; M, male; TLE, temporal lobe epilepsy; uncl. IGE, unclassified idiopathic generalized epilepsy; FLE, frontal lobe epilepsy; HS, hippocampal sclerosis; CD, celiac disease; IgA, immunoglobulin A; juvRA, juvenile rheumatoid arthritis; T1D, type 1 diabetes; S, serum; IS, intrathecal synthesis; RIA, radioimmunoassay; TPO, thyroperoxidase antibody; GM1, GM1 antibody; LANG, Langerhans cell antibody; TYGL, thyroglobulin antibody; ANA, antinuclear antibody; b2GP1, b2-glycoprotein 1 antibody; AGPC, antigastric parietal cell antibody. Updated and modified from our previous publication (Liimatainen et al., 2010). IL-6 and IL-10 levels and their ratio are presented as median values in the table.

### 3.2. Plasma IL-6 and IL-10 concentrations and their ratio

**FIGURE 1:**
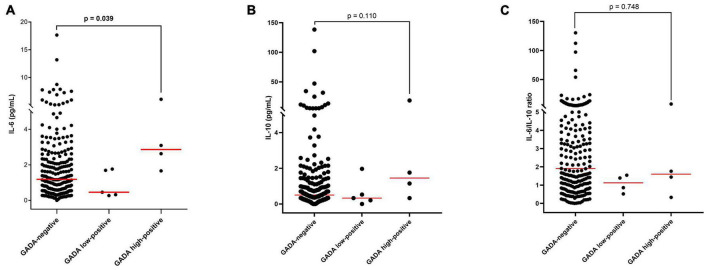
Distribution and median concentrations of IL-6 and IL-10 levels and their ratio in different study groups: **(A)** IL-6 levels, **(B)** IL-10 levels, and **(C)** the IL-6/IL-10 ratio.

Median IL-6 levels were significantly higher in the high GADA positive group than in GADA-negative group (*p* = 0.039, Figure 1A). Similarly, IL-10 levels were also higher in the GADA high-positive group than in the GADA-negative group, however, the difference was not statistically significant (*p* = 0.110, Figure 1B). The difference in the IL-6/IL-10 ratio between these two groups was similar (Figure 1C). The median (IQR) IL-6 levels for the GADA-negative vs. GADA high-positive groups were 1.18 (0.54–2.32) vs. 2.86 (1.90–5.34), and the median (IQR) IL-10 levels were 0.50 (0.24–1.00) vs. 1.45 (0.53–14.32). IL-6 and IL-10 levels and their ratio did not change either between GADA-negative and GADA low-positive groups or between the GADA low-positive and GADA high-positive groups (*p* > 0.05). The distribution of IL-6 and IL-10 levels and their ratio in each group are shown in Figures 1A–C.

Moreover, we made subgroups of patients based on their types of epilepsy, etiologies, and abnormal MRI, and compared the IL-6 and IL-10 levels and their ratio, in different GADA groups. The results showed no significant difference between the groups based on epilepsy types (*p* > 0.05, Supplementary Figures 1A–C), between the groups based on etiologies (*p* > 0.05, Supplementary Figures 2A–C), and between the groups based on abnormal MRI (*p* > 0.05, Supplementary Figure 3A–C). Similarly, we found no significant difference in IL-6 and IL-10 levels and their ratio in subgroups of patients based on with or without autoimmune diseases (*p* > 0.05, Supplementary Figures 4A–C).

We also performed the correlation analysis between the IL-6 and IL-10 levels and their ratio with the seizure frequency during the last month before the lab and the results showed no significant association between the cytokines and their ratio with the seizure frequency in all GADA groups (Supplementary Figures 5A–I).

## 4. Discussion

The present study assessed the relationship between levels of the soluble cytokines IL-6 and IL-10, including the IL6/IL10 ratio and GADA titers, in patients with DRE in the same population that had GADA titers measured previously to evaluate their clinical significance in epilepsy (Liimatainen et al., 2010). The results from our present study showed elevated levels of IL-6 in patients who were highly positive for GADA compared to patients who were GADA negative. Moreover, IL-10 levels were also higher in GADA high-positive patients than in GADA-negative patients but lacked a statistically significant difference. The IL-6/IL-10 ratio remained unaltered among all groups.

Considering the critical role of IL-6-mediated inflammation in autoimmune neurological diseases, increased IL-6 levels in the GADA high-positive group may indicate a role for IL-6 in initiating, and propagating autoimmune inflammation in our patients (Moudgil and Choubey, 2011). Moreover, unchanged levels of IL-10 among GADA groups suggest that IL-10 could not contribute adequate anti-inflammatory supply to counteract the excessive inflammation driven by IL-6. In line with this observation, we recently detected a significant reduction in IL-10 in TLE with HS (Basnyat et al., 2020). Similarly, the altered levels of IL-6 and IL-10 in different GADA groups also indicate a Th1/Th2 cytokine imbalance, a representative phenomenon observed in patients with T1DM (Ng et al., 1999).

Although comparing the immunopathogenesis of T1DM is irrelevant to epilepsy, an increased risk of epilepsy has been reported in patients with autoimmune diabetes (Dafoulas et al., 2017; Yan et al., 2017). A similar positive association has been previously reported between systemic inflammation, i.e., increased serum cytokine IL-1 β, IL-3, INF- γ, and GADA levels in patients with both types of diabetes (Amin et al., 2020) and significantly higher IL-6 and IL-15 concentrations in autoimmune diabetes (Siewko et al., 2019). Thus, the predominance of the GAD-specific antibody response along with systemic inflammation suggest that IL-6 signaling could be a novel pharmacological target in the treatment of GADA-induced autoimmune epilepsy. Several drugs, such as anakinra (human recombinant IL-1Ra), tocilizumab (a monoclonal antibody against the IL-6 receptor) and reparixin (an IL-8 receptor inhibitor), have been suggested as possible drugs to prevent epilepsy (Vezzani et al., 2019). Nonetheless, dual biological components, high GADA titers and elevated systemic levels of IL-6, should be evaluated further for their biomarker potential for the assessment of GADA-induced autoimmune epilepsy and as one of the possible anti-inflammatory interventions in its treatment. We previously reported the significance of early detection of GAD65 antibodies in the immunotherapy treatment of patients with DRE (Mäkelä et al., 2018).

Interleukin-6 not only induces acute phase reactions but also induces autoantibody production, such as anti-aquaporin 4 autoantibody production in neuromyelitis optica (Chihara et al., 2011). In this regard, the ability of IL-6 to induce B-cell differentiation could result in the secretion of several pathological autoantibodies, probably including GADA. Additionally, IL-6 affects T cells by enhancing overreactive Th1 activity, the mechanism responsible for the induction of several organ-specific autoimmune inflammatory diseases, such as multiple sclerosis, rheumatoid arthritis and T1DM (Moudgil and Choubey, 2011). Likewise, excessive and continuous production of IL-6 during epileptic seizures could be the cause of GADA production. Previously, we found that approximately 90% of patients who had high GADA titers also had TLE, and the immunologic profile of these patients suggested a possible autoimmune etiology (Liimatainen et al., 2010). An experimental study reported CNS destruction with lethal encephalomyelitis-like disease mediated by GAD-specific CD4^+^ T cells; conversely, the effect of B-cell-generated high titer anti-GAD65 autoantibodies had no effect on the incidence or severity of disease (Burton et al., 2010). In addition, GADA high-positive epilepsy patients in our study also had a higher number of other autoimmune diseases than patients without GADA. Furthermore, the number of other autoantibodies was also higher in GADA high-positive patients, as reported previously (Liimatainen et al., 2010), which may signify the existence of a poly autoimmune etiology of epilepsy. Along these lines, our findings support the idea that IL-6-mediated systemic inflammation could also be the cause for the presence of high titers of GADA.

On the other hand, IL-6-mediated systemic inflammation could also be the result of the presence of GADA. The pathogenic significance of GADA in neuroinflammation is complex (McKeon et al., 2012; Toledano et al., 2014). Epilepsy-specific antibodies such as NMDA receptor (NMDAR) and voltage-gated potassium channel (VGKC) complex antibodies are regarded as directly pathogenic to the brain (Liimatainen et al., 2014). Previously, in patients with high GADA titers, we found intrathecal synthesis of GADA, suggesting that GADA could be a marker of an ongoing immune response (Liimatainen et al., 2010), and in the same patients, we found high levels of IL-6. Thus, this finding may support the role of GADA in the pathogenesis of epilepsy, and elevated IL-6 levels in the same patients could reveal a further immunological basis.

Some studies have suggested that GADA is not pathogenically active by itself but only an indicator of the immunopathological process (Bien and Scheffer, 2011), whereas other findings have supported its pathogenicity (Petroff et al., 1996; Mitoma et al., 2000; Vianello et al., 2008; Munoz-Lopetegi et al., 2020). GAD autoimmunity could induce epileptogenic activity and increases seizure susceptibility thus initiating the activation of inflammatory pathways, both in the periphery and in the CNS, resulting in the higher production of proinflammatory cytokines such as IL-6 (Mitoma et al., 2000). Therefore, these explanations may fairly support that IL-6-mediated systemic inflammation could be the consequence of high GADA titers. It is also important to consider that IL-6 production is triggered by acute epileptic seizures (Alapirtti et al., 2018), increasing the likelihood that they may be causal rather than the consequence of autoimmune aspects of epilepsy.

Moreover, high titers of peripheral GADA upon systemic inflammation, but not low titers, can cross the blood-brain barrier mediating CNS damage, as in the case of SPS, and should be evaluated in epilepsy as well (Ali et al., 2011). Therefore, high IL-6 production and high GADA titers could be the dual components attributable to the genesis of autoimmune epilepsy. Moreover, since we lack a broader interpretation of our second hypothesis, a future study is warranted with a simultaneous measurement of IL-6 and GADA in a new set of patients.

One of the main limitations of our study is the low number of patients and the retrospective nature of the study design, which makes it difficult to draw definite conclusions. However, GADA-associated epilepsy is a rare entity, and a larger sample size is implausible. In addition, due to limited access, we were unable to perform cytokine measurements in the CSF samples.

Taken together, the results of the present study may provide additional pathophysiologic significance of IL-6 in GADA-associated autoimmune epilepsy. It also offers the new possibility that IL-6 signaling could be a promising anti-inflammatory therapeutic intervention in the treatment of GADA-induced autoimmune epilepsy. However, future research is warranted to elucidate whether GADA are pathogenic entities or only serve as surrogate markers for autoimmune diseases mediated by cytotoxic T cells.

## Data availability statement

The raw data supporting the conclusions of this article will be made available by the authors, without undue reservation.

## Ethics statement

The studies involving human participants were reviewed and approved by the Ethics Committee of Tampere University Hospital. The patients/participants provided their written informed consent to participate in this study.

## Author contributions

PB and MPel analyzed the data and wrote the manuscript with input from all authors. JR performed statistical analyses. JP, MPes, SL, and SR conceived the study design. JP and MPes supervised the research. All authors contributed to the article and approved the submitted version.
