# Supplementary material for: Elevated IL-6 plasma levels are associated with GAD antibodies-associated autoimmune epilepsy
Source: Front Cell Neurosci. 2023 Mar 21;17:1129907. doi: 10.3389/fncel.2023.1129907 (PMC10070787; doi:10.3389/fncel.2023.1129907)
Supplement: Supplementary file 1 [file Data_Sheet_1.pdf]

Supplementary results

Epilepsy types: There were no statistically significant difference in IL-6, IL-10 or IL-6/IL-10 ratio among different GADA groups based on epilepsy types ($p>0.05$, Kruskal Wallis Test).

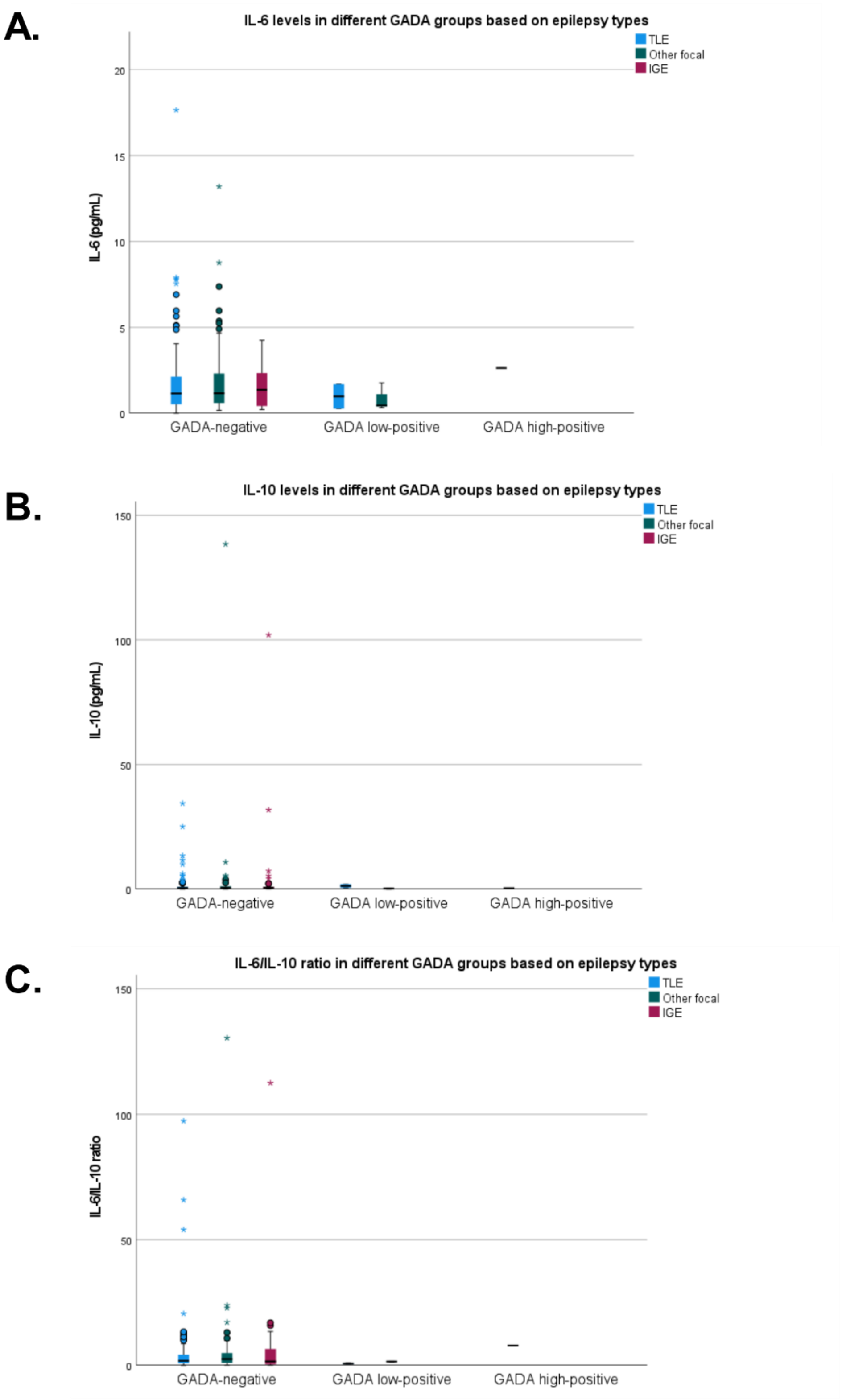

Supplementary Fig 1A-C. Boxplots for IL-6, IL-10 and their ratio in subgroups based on epilepsy types.

Etiologies: There were no statistically significant difference in IL-6, IL-10 or IL-6/IL-10 ratio among different GADA groups based on etiologies (p>0.05, Kruskal Wallis Test).

A.

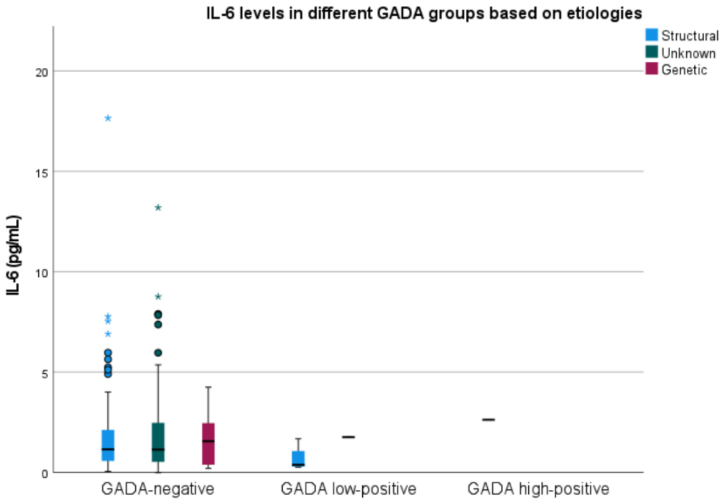

B.

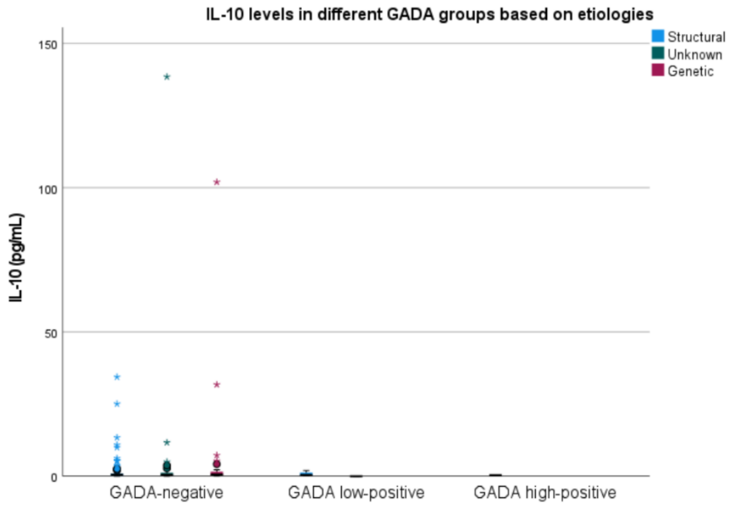

C.

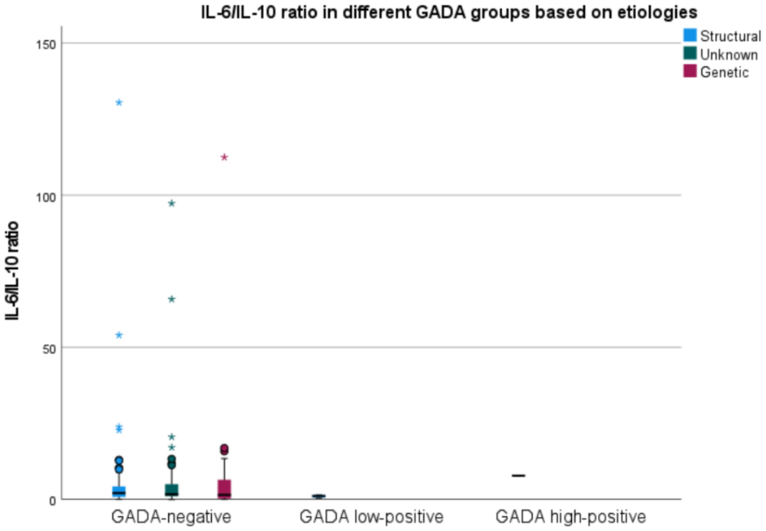

Supplementary Fig 2A-C. Boxplots for IL-6, IL-10 and their ratio in subgroups based on etiologies.

MRI abnormal (yes/no): There were no statistically significant difference in IL-6, IL-10 or IL-6/IL-10 ratio among different GADA groups based on abnormal MRI (p>0.05, Kruskal Wallis Test).

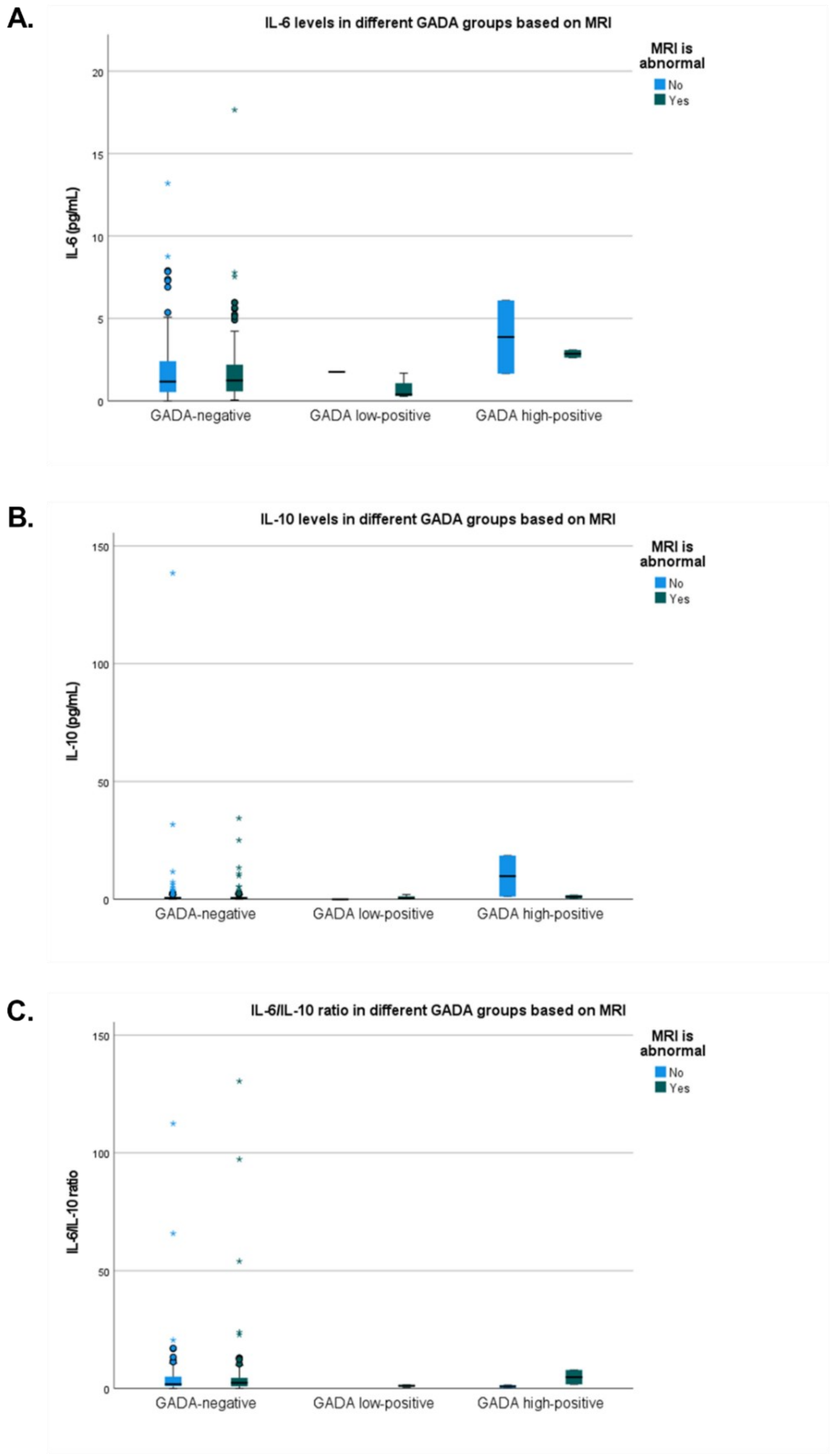

Supplementary Fig 3A-C. Boxplots for IL-6, IL-10 and their ratio in subgroups based on abnormal MRI

With or without autoimmune diseases: There were no statistically significant difference in IL-6, IL-10 or IL-6/IL-10 ratio among different GADA groups of patients with or without autoimmune diseases (p>0.05, Kruskal Wallis Test).

A.

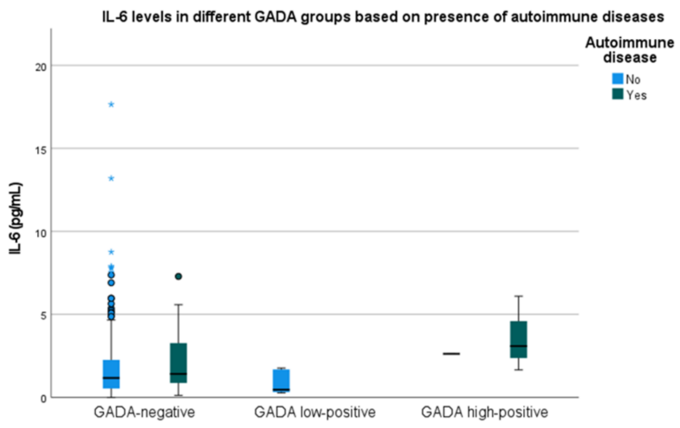

B.

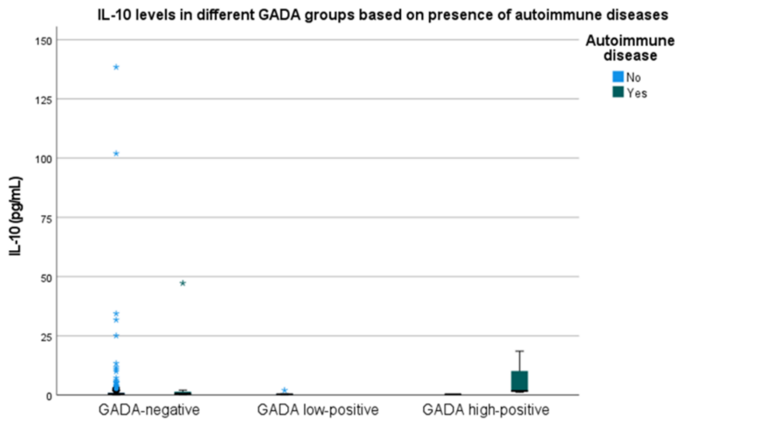

C.

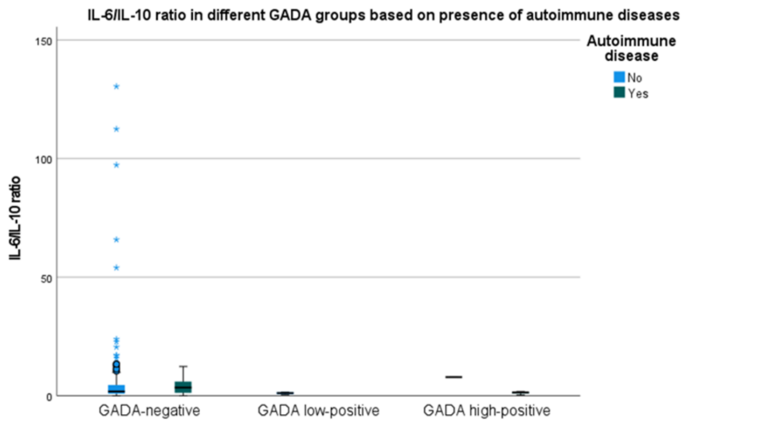

Supplementary Fig 4A-C. Boxplots for IL-6, IL-10 and their ratio in subgroups based on patients with or without autoimmune diseases.

Spearman's Correlation analysis between IL-6, IL-10 and their ratio with seizure frequency during the last month before the lab. There were no significant association between the cytokines and their ratio with the seizure frequency in all GADA groups.

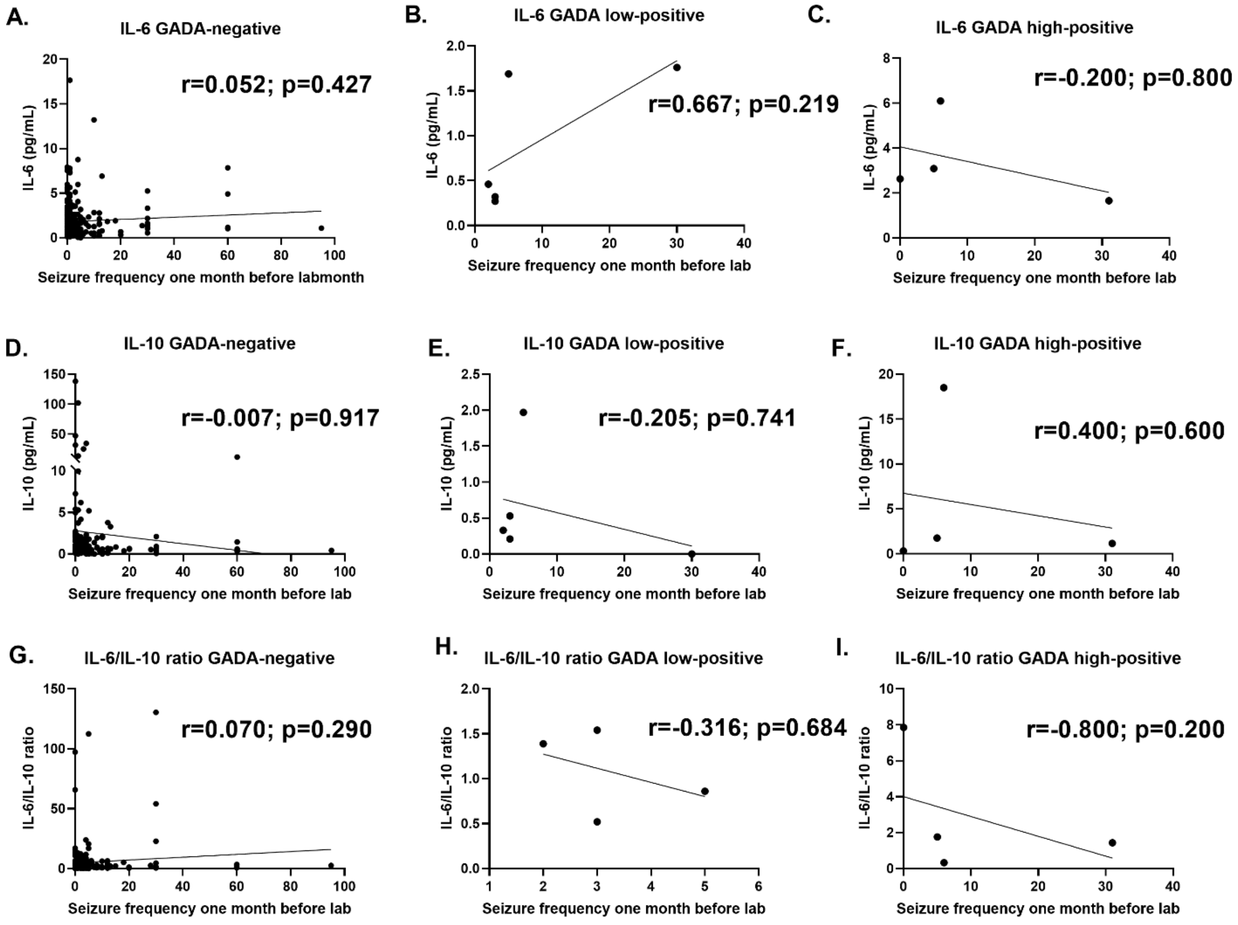

Supplementary Fig 5A-I. Scatter plots for correlation between IL-6, IL-10 and their ratio with the seizure frequency during the last month before lab.
